# Molecular Immunology in Hematological Disorders

**DOI:** 10.3390/ijms23179584

**Published:** 2022-08-24

**Authors:** Akiyoshi Takami

**Affiliations:** Department of Medicine, Division of Hematology, Aichi Medical University School of Medicine, Nagakute 480-1195, Japan; takami-knz@umin.ac.jp

This Special Issue aims to highlight the molecular mechanisms involved in the development and progression of hematologic malignancies such as leukemia, lymphoma, and myeloma. The fact that hematologic cancer cells can be eradicated not only by allogeneic hematopoietic stem cell transplantation, but also by single-molecule targeted therapies, such as chimeric antigen receptor (CAR)-T cells, suggests that the immune system, including innate and adaptive immunity, plays an important role in hematologic malignancies. Such immune therapies have also proven successful in non-cancerous hematologic conditions such as thrombotic microangiopathy and hemolytic anemia. Furthermore, hematologic abnormalities associated with immunological dysregulation have been observed in several infectious diseases, such as COVID-19, and autoimmune diseases as well. Therefore, there is a close pathophysiological relationship between hematological diseases and the immune system, and the elucidation of these molecular mechanisms will advance the diagnosis and treatment of benign and malignant hematologic, infectious, and autoimmune diseases. Contemporary studies [1,2,3,4,5,6] have focused on the applications of molecular immunology in hematology, providing fresh insights into the molecular immunological mechanisms involved in the diagnosis and treatment of hematologic diseases.

Although allogeneic hematopoietic stem cell transplantation can effectively treat leukemia through the graft-versus-leukemia (GVL) effect, it is currently difficult to avoid conditioning-related toxicity, severe infections, graft-versus-host disease (GVHD), and their associated mortality and morbidity [7,8,9,10,11,12]. Observations made by Espinoza et al. [6] may overcome this dilemma. The study showed that the gene encoding the NLR family pyrin domain-containing 3 (NLRP3) protein, which plays pivotal roles in innate immune responses, exhibits a functional polymorphism, and transplant recipients with the *NLRP3* gene polymorphisms that overexpress NLRP3 were more likely to induce the production of IL-1b than other transplant recipients, leading to increased transplant-related mortality. These findings suggest that conditioning-induced tissue injury and infection lead to an overexpression of IL-1b due to the overexpression of NLRP3, leading to transplant-related mortality, because post-transplant IL-1b overexpression induces and exacerbates post-transplant organ toxicity and severe GVHD, independent of GVL effects [13]. Therefore, the development of molecular agents that target functional polymorphisms in the *NLRP3* gene may prevent post-transplantation organ damage and mortality.

For developing antigen-specific immunotherapies, such as CAR-T cell therapy and molecular-targeted agents against leukemia, antigens that are expressed only in leukemia cells and not in normal tissues (e.g., the *BCR-ABL* chimeric gene which produces antigens specific to chronic myeloid leukemia) are the ideal targets. However, leukemia-associated antigens that are expressed in normal tissues, but overexpressed in leukemia cells (such as the CD19 antigen in acute lymphocytic leukemia and CD33 antigen in acute myeloid leukemia), can also be therapeutic target antigens, although normal blood cells, as well as leukemic cells, are eradicated in such cases. There are few leukemia-specific antigens with known utility against acute myeloid leukemia; hence, clinically available CAR-T cell therapies against it currently do not exist. Forghieri et al. [5] summarized that neoantigens, such as peptides derived from *NPM1* gene mutant proteins that are not present in normal human tissues, can be considered to be leukemia-specific antigens. *NPM1* gene mutations are the most common mutations (30%) in acute myeloid leukemia. *NPM1* gene mutations are considered a favorable prognostic factor for acute myeloid leukemia; however, relapse rates reach 50% to 60%. Furthermore, *NPM1* mutations tend to coexist with gene mutations with poor prognoses, such as *FLT3-ITD* mutations, and, in such cases, the prognosis of acute myeloid leukemia is poor. Consequently, research on these gene mutations is important, because they may lead to specific and effective molecular-targeted therapies for acute myeloid leukemia, such as CAR-T cell therapy and peptide vaccine therapy.

Cytokine release syndrome (CRS) and immune-effector-cell-associated neurotoxic syndrome (ICANS) are immune-mediated toxicities characterized by the overexpression and overactivation of proinflammatory cytokines, among which TNF-α and IL-1b play crucial roles. Although these complications have been found to hinder the success of CAR-T cell therapy, it was unclear whether ICANS occurred after allogeneic hematopoietic stem cell transplantation. Marcuzzi et al. [3] found that three pediatric patients treated with donor lymphocyte infusion for immunodeficiency-associated viral infections (Epstein–Barr virus in two, adenovirus in one) after allogeneic hematopoietic stem cell transplantation developed CRS and ICANS with increased TNF-α and IL-1b. Moreover, their study showed that treatment with the TNF-α inhibitor infliximab was effective in all three cases of ICANS. These findings indicate that the large quantities of T lymphocytes infused after allogeneic hematopoietic stem cell transplantation recognized the virus-associated antigens, and rapidly activated and released large amounts of proinflammatory cytokines such as TNF-α and IL-1b. This may be similar to the pathogenesis in which CAR-T cells recognize tumor-associated antigens and cause CRS and ICANS. The mechanism through which T cell infusion causes ICANS remains unclear. However, the clinical observations of this study provided insight into the pathogenesis and treatment of ICANS, and suggested that the occurrence of ICANS after allogeneic hematopoietic stem cell transplantation may be underestimated.

One of the breakthroughs in the treatment of hematologic malignancies over the past several decades includes the establishment of molecular-targeted therapy for multiple myeloma (MM). However, single targeted drug therapy is unlikely to induce durable molecular remission, suggesting that myeloma cell clones may acquire immune evasion and cell proliferation throughout the clinical course. This may be supported by the fact that the monoclonal gammopathy of undetermined significance (MGUS) can progress to smoldering MM and active MM. Although the mechanisms through which myeloma cell clones evade the immune system are not well understood, it has been suggested that impaired T cell function may inhibit the eradication of myeloma cell clones. Lagreca et al. [4] provide an excellent review, focusing on the role of T cells and the bone marrow microenvironment in the development and progression of MM. Myeloma cell clones are assumed to acquire genetic abnormalities that favor proliferation and immune escape. These genetic abnormalities promote myeloma clone proliferation by disturbing the immune network in the bone marrow microenvironment and suppressing myeloma-specific T cell immune surveillance. Conversely, the restoration of such functional anti-myeloma T cell immunity may not only prevent the progression of MGUS to MM, but also provide an effective therapeutic option for refractory MM. As suggested by this study, clarifying the immunopathology and immunological biomarkers in MM will lead to further advances in the treatment of MM.

Nakamura et al. [2] demonstrated that the BCL-2-selective inhibitor venetoclax synergistically enhances the anti-MM effects of the anti-CD38 antibody drug daratumumab. This not only indicated that the combination therapy of venetoclax and daratumumab could be a therapeutic option for refractory MM, but also suggested that mitochondria-targeted therapy might be useful for MM, because venetoclax primarily acts on mitochondria.

The COVID-19 pandemic, i.e., the global outbreak caused by severe acute respiratory syndrome coronavirus 2 (SARS-CoV-2), remains one of the greatest contemporary public health concerns. COVID-19 infections result in both coagulation and fibrinolytic activation, which can lead to thrombosis and hemorrhage, respectively. Similarly, for SARS-CoV-2 vaccination, vaccine-induced immune thrombotic thrombocytopenia (VITT) and vaccine-induced hyperfibrinolytic disseminated intravascular coagulation have been reported. Therefore, insight and knowledge regarding both thrombosis and hemostasis are required for COVID-19 care. Yamada et al. [1] effectively summarized the pathophysiology, treatments, and contraindications for coagulopathy, thrombosis, and bleeding disorders caused by both COVID-19 and SARS-CoV-2 vaccination. These findings have important implications for the treatment of SARS-CoV-2 infection, and provide useful insight for medical care related to COVID-19.

## Data Availability

Not applicable.
